# Preparation of Multilayer Platforms for Advanced Wound Care Management

**DOI:** 10.3390/polym17172393

**Published:** 2025-09-02

**Authors:** Amir Mohammad Sharafi, Sina Pakkhesal, Farnaz Monajjemzadeh, Nastaran Alipour, Samin Hamidi

**Affiliations:** 1 Student Research Committee, Tabriz University of Medical Sciences, Tabriz, Iran; sharafiamirmohammad@gmail.com (A.M.S.); sinapak1380@gmail.com (S.P.); 2 Department of Pharmaceutical and Food Control, Faculty of Pharmacy, Tabriz University of Medical Sciences, Tabriz, Iran; monajjemzadehf@yahoo.com; 3 Drug Applied Research Center, Department of Medical Nanotechnology, Faculty of Advanced Medical Sciences, Tabriz University of Medical Sciences, Tabriz, Iran; nastaranalipoure@gmail.com; 4 Research Center of Psychiatry and Behavioral Sciences, Tabriz University of Medical Sciences, Tabriz, Iran

**Keywords:** advanced wound care, bioengineering, multilayer platforms

## Abstract

Multilayer platforms have emerged as promising tools in the field of wound healing, offering a multifaceted approach to promote effective and accelerated tissue regeneration. This review article aims to provide a comprehensive overview of the various multilayer platforms employed in wound healing applications, highlighting their structure, fabrication methods, and potential mechanisms of action. The first section of the review focuses on the design and composition of multilayer platforms, encompassing different materials such as polymers, hydrogels, and biocompatible scaffolds. It discusses the significance of each layer in terms of its specific functionalities, including cell adhesion, drug/bioactive factor loading, antimicrobial properties, and mechanical support. The second section of the review delves into the mechanisms of action associated with multilayer platforms in wound healing. It discusses how these platforms facilitate wound closure, promote angiogenesis, modulate inflammation, and enhance tissue regeneration. The article also examines the role of multilayer platforms in providing a physical barrier against external pathogens, reducing the risk of infection, and creating a favorable microenvironment for wound healing. Overall, this review highlights the significant advancements made in the field of multilayer platforms for wound healing and underscores their potential as versatile therapeutic strategies.

## 1. Introduction

Skin and wound care encompasses the management of disturbances in the customary configuration and operation of the skin and the architectural framework of soft tissues [1]. The skin, being the largest organ of the body, necessitates a profound comprehension of its composition and functions for effective wound care. Structurally, the skin comprises three layers: the epidermis, dermis, and hypodermis [2]. The epidermis, occupying the outermost position, comprises non-living cells that undergo constant exfoliation. The dermis, positioned in the middle, consists of connective tissue, blood vessels, nerves, and hair follicles. Lastly, the hypodermis, the deepest layer of the skin, consists predominantly of adipose cells [3]. Wound healing is an intricate and multifaceted procedure comprising distinct stages. This process involves three interconnected phases, namely inflammation, proliferation, and remodeling. Any disruption incurred during these phases results in aberrant wound healing [4]. Wounds can be categorized based on their severity and anatomical location. Superficial wounds encompass those solely affecting the epidermal layer [5], while partial-thickness wounds involve impairment to both the epidermis and dermis [6]. Lastly, full-thickness wounds encompass injuries that extend through the epidermis, dermis, and hypodermis layers [7]. Moreover, wounds can also be classified based on the progression and duration of healing, distinguishing them as acute or chronic [8]. Additional classification methods for wounds exist, pertaining to their manner or modality [9].

Acute wounds are a type of injury that typically occurs suddenly and heals relatively quickly within 4 weeks. They can be caused by various mechanisms, including trauma, surgery, or burns [10]. Acute wounds are characterized by an inflammatory response that helps clear debris and bacteria from the wound site and stimulates new tissue growth [9,10]. This process involves the release of cytokines and growth factors, which help to recruit immune cells and promote angiogenesis and collagen synthesis [11]. Treatment for acute wounds typically involves cleaning, debriding if necessary, and dressing to promote healing. In some cases, antibiotics may be prescribed to prevent infection [12]. Properly managing acute wounds prevents complications such as delayed healing, infection, or scarring [13].

Chronic wounds are injuries that persist for an extended period and fail to heal promptly [14]. They can be caused by various underlying conditions, such as diabetes or vascular disease, which impair the normal wound-healing process [9,14]. Chronic wounds are characterized by persistent inflammation, increased levels of proteases, and decreased levels of growth factors, which all contribute to impaired tissue regeneration [15]. Treatment for chronic wounds typically involves addressing the underlying condition, debridement if necessary, and dressings to promote healing. In some cases, advanced therapies such as negative pressure wound therapy or bioengineered skin substitutes may be used [12]. Properly managing chronic wounds is important to prevent complications such as infection or amputation [16].

Multilayer platforms have exhibited considerable potential in facilitating the process of wound healing [17]. Through the incorporation of diverse layers composed of materials possessing distinctive properties, these platforms offer a range of advantages for the healing process [18]. Specifically, they can provide mechanical reinforcement to the wound site along with the delivery of therapeutic agents, including growth factors and antimicrobial compounds [18,19,20,21,22]. These dressings consist of multiple layers working in harmony to establish favorable conditions for healing, such as moisture regulation, safeguarding against infection, and stimulation of tissue regeneration [17,19,23]. Furthermore, multilayer platforms can be engineered to enhance the migration and proliferation of cells, pivotal stages in the sequence of wound healing events [24]. Hydrogels, nanofibers, and microparticles are among the materials suitable for integration into multilayer platforms [20,25,26,27,28]. These materials can be tailored to exhibit specific properties like biocompatibility, degradation rate, and drug release kinetics [17,19,29,30]. Multilayer wound dressing platforms have emerged as a promising strategy for addressing both acute and chronic wounds. In the case of acute injuries, multilayer dressings serve as a barrier between the wound and the external environment, thereby preventing complications such as delayed healing and infection [31]. For chronic wounds, these dressings target underlying factors that impede normal wound healing, such as inflammation [32] and protease activity [33]. Moreover, multilayer dressings can be designed to release bioactive agents that facilitate tissue regeneration or exhibit antimicrobial properties [34]. In summary, multilayer wound dressing platforms offer significant potential in enhancing outcomes in the management of both acute and chronic wounds. They represent a captivating avenue for achieving improved outcomes in the realm of wound healing.

## 2. Processes Governing Wound Healing

Cutaneous wound healing involves interconnected and complex events by cells, proteins, enzymes, extracellular matrix components, and growth factors [35]. This process is basically divided into three general stages. However, this division is not meant as a sequential process, and due to the difference of dominant cells in these phases, a considerable amount of overlap can be expected. The three principal stages of the wound healing process include 1. inflammatory phase; 2. proliferative phase; and 3. maturational phase (tissue remodeling) [36].

The inflammatory phase is the fastest stage of wound healing, in which hemostasis and inflammation-associated events occur. Hemostatic events are triggered by epithelial cell damage and collagen exposure, starting with the clotting cascade. Also, this damage leads to prostaglandin 2-alpha and thromboxane A2 release, which cause intense vasoconstriction and amplification of platelet activation, respectively [36]. Platelets play a crucial role in all processes of the inflammatory phase, especially hemostatic plug formation and chemokine secretion [37]. The initial raised responses control the bleeding and then prepare an extracellular matrix, consequently supporting cell migration. Following the hemostasis attained, the complement cascade activated by platelet degranulation results in histamine release. Histamine alters vascular permeability, allowing the inflammatory and phagocyte cells to enter and migrate to the wound site. Whereby mentioned cell activities lead to the lysis of bacteria, engulf dead cells, liberate platelets, and form aggregates [36,38].

The proliferative phase is summarized in four main events: epithelialization, granulation, angiogenesis, and collagen deposition [39]. In a brief explanation, the epithelial cells move upwards during epithelialization to repair the damaged area. Granulation refers to the function of macrophages and fibroblasts to release various growth factors and factors stimulating the formation of fibrous tissue (fibroplasia). Angiogenesis increases the nutrient supply needed for the healing process in the area. Insufficiency of the provided supply causes chronic wounds associated with diseases such as diabetes. In the last term, collagen deposition caused by fibroblasts’ activities fills the defect [39,40].

**Figure 1:**
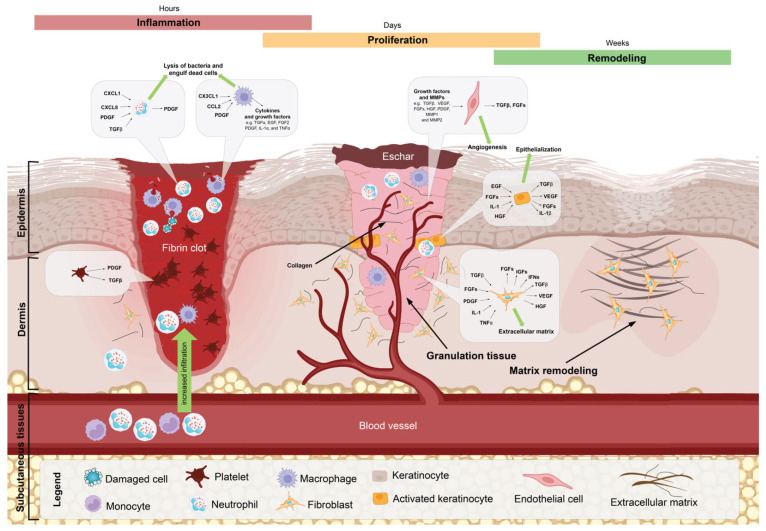
Cellular and Molecular Overview of the Wound Healing Process in Three Stages: Inflammation, proliferation, and remodeling: This figure illustrates the complex, multi-phase process of wound healing, encompassing three distinct stages: inflammation, proliferation, and remodeling, each characterized by precise cellular and molecular events that facilitate tissue repair and restoration of structural integrity. (1) Inflammation Phase (Hours): Upon tissue injury, the wound environment undergoes immediate hemostasis, forming a fibrin clot to prevent further blood loss and establish a provisional extracellular matrix (ECM) for subsequent cellular infiltration. Platelets release potent growth factors, including Platelet-Derived Growth Factor (PDGF) and Transforming Growth Factor-Beta (TGF-β), which orchestrate the recruitment of neutrophils and macrophages. Neutrophils, depicted as blue cells, are the first responders to the wound, engaging in phagocytosis of debris and bacteria while releasing pro-inflammatory cytokines like CXCL1, IL-1, and TNF-α. As neutrophils clear pathogens, macrophages (purple cells) succeed them, continuing the clearance of necrotic debris and releasing additional cytokines (e.g., CCL2) to further recruit and activate fibroblasts and endothelial cells. This inflammatory milieu is essential for the transition to the next phase. (2) Proliferation Phase (Days): In the proliferative phase, granulation tissue forms beneath the eschar. Fibroblasts (orange cells) are highly active in synthesizing collagen and ECM components. The angiogenic process, critical for oxygen and nutrient supply to the healing tissue, is driven by Vascular Endothelial Growth Factor (VEGF) and Fibroblast Growth Factors (FGFs), which stimulate the proliferation of endothelial cells and the formation of new capillaries. Simultaneously, keratinocytes migrate from the wound edges, a process known as re-epithelialization, which is mediated by factors such as Epidermal Growth Factor (EGF), Hepatocyte Growth Factor (HGF), and TGF-β. The formation of granulation tissue, characterized by its dense cellular composition and new vasculature, provides a scaffold for ongoing ECM deposition and wound closure. (3) Remodeling Phase (Weeks): The final remodeling phase is marked by ECM maturation and reorganization. Fibroblasts continue to secrete collagen, specifically collagen type I, which replaces the initial collagen type III to increase tensile strength. Growth factors like TGF-β, FGFs, and PDGF modulate this process, while matrix metalloproteinases (MMPs) play a critical role in ECM degradation and turnover, balancing collagen synthesis with degradation to prevent fibrosis. The extracellular matrix is progressively remodeled to resemble the pre-injury dermal architecture, restoring skin integrity. Blood vessels regress and fibroblasts differentiate into myofibroblasts, which contract the wound, reducing its size and aiding closure. Interleukin-1 (IL-1); interleukin-6 (IL-6); tumor necrosis factor-alpha (TNF-α); transforming growth factor-beta (TGF-β); interleukin-10 (IL-10); platelet-derived growth factor (PDGF); fibroblast growth factor (FGF); extracellular matrix (ECM); matrix metalloproteinases (MMPs); hepatocyte growth factor (HGF); and insulin-like growth factors (IGFs).

The maturational phase (also the tissue remodeling phase) refers to the sum of all the events that cause the shift from granulation tissue to scar tissue [41]. Depending on the injury, as well as factors like the location and the extent of the injury, this stage starts a few days after the injury, and the continuation time can vary from a few days to several months. In this phase, the main activities in wound healing are performed by proteases and fibroblasts (with the function of collagen deposition); contraction of the size of the wound, the amount of scar left behind, and scar formation are the final results of the actions performed during this phase [42,43]. Figure 1 shows the complex interplay of cellular and molecular events during the three major stages of wound healing.

Figure 1 illustrates the complex interplay of cellular and molecular events during the three major stages of wound healing: inflammation, proliferation, and remodeling. The process begins with the inflammatory stage, characterized by hemostasis and the formation of a fibrin clot, which serves as a temporary barrier to prevent blood loss and infection. Upon injury, cells at the wound site release pro-inflammatory cytokines such as interleukin-1 (IL-1), interleukin-6 (IL-6), and tumor necrosis factor-alpha (TNF-α). These cytokines stimulate the recruitment of immune cells, including neutrophils and macrophages, to the wound area. During this stage, various immune cells, such as neutrophils and macrophages, are recruited to the wound site to clear debris, remove pathogens, and release cytokines and growth factors that initiate the healing process. Cytokines like transforming growth factor-beta (TGF-β) and interleukin-10 (IL-10) possess anti-inflammatory properties and help resolve inflammation. They also play a role in facilitating the transition from the inflammatory phase to the proliferative phase of wound healing by promoting fibroblast and endothelial cell activation. The proliferation stage follows, marked by the activation of fibroblasts, endothelial cells, and keratinocytes. Cytokines, along with growth factors, are responsible for the migration and proliferation of various cell types involved in wound healing. For example, platelet-derived growth factor (PDGF) and fibroblast growth factor (FGF) promote fibroblast migration and proliferation, while vascular endothelial growth factor (VEGF) stimulates angiogenesis by promoting endothelial cell migration and proliferation. Fibroblasts produce extracellular matrix (ECM) components, such as collagen and fibronectin, and differentiate into myofibroblasts, which contribute to wound contraction. Cytokines are involved in the regulation of myofibroblast differentiation, apoptosis, and scar maturation. For example, TGF-β promotes the differentiation of fibroblasts into myofibroblasts, which are responsible for wound contraction. The balance between pro-inflammatory and anti-inflammatory cytokines also influences the extent of scar formation and tissue remodeling. Endothelial cells form new blood vessels through angiogenesis, ensuring adequate oxygen and nutrient supply to the healing tissue. Keratinocytes migrate and proliferate at the wound edges to re-epithelialize the injured site. Cytokines like TGF-β play a critical role in ECM deposition by stimulating the synthesis of matrix components such as collagen, fibronectin, and proteoglycans. They also regulate the balance between matrix metalloproteinases (MMPs) and their inhibitors, which is crucial for the proper remodeling of the ECM during wound healing. Finally, the remodeling stage takes place, during which the provisional ECM is replaced by a more organized and mature matrix. Collagen fibers become more densely packed, and the ratio of collagen type III to type I increases, providing greater tensile strength to the repaired tissue. Myofibroblasts undergo apoptosis, and blood vessels regress, resulting in a reduction in vascular density. This stage can last for months or even years, depending on the size and severity of the wound.

## 3. Traditional Wound Dressings

### 3.1. Primary Wound Care

**Figure 2:**
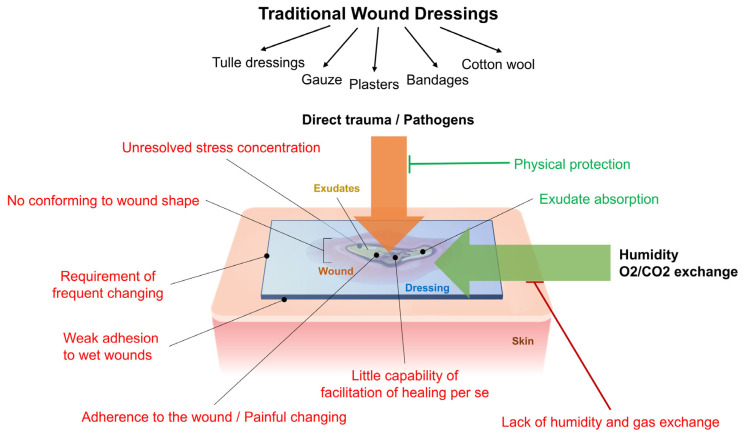
Traditional wound dressing.

Different substances and materials have been used in medical care as wound dressings, including bandages, gauze, cotton wool, lint, oakum (shredded rope), etc. [44,45]. The primary purpose of this dressing was to absorb exudate and provide physical protection against pathogens. To put it another way, the purpose of wound management consisted of ‘to cover and conceal’. These materials had little impact on the wound healing process [46]. The lack of humidity caused traditional materials to be replaced with advanced and novel devices, which were highly regarded due to the provision of humidity, effective gas exchange, prevention of microbial contamination and colonization, and the accumulation of exudate [46]. However, traditional dressings are limited in application due to sticking to the wound site and causing pain when removed [47,48]. Due to evaporation, gauze dressings made of cotton and polyester fibers cause a lack of water in the wound and thus delay the wound-healing process. Also, they may loosen and sink into the wounded skin, which causes discomfort to the patient [49]. In conclusion, traditional wound dressings are easy to apply, low-cost, and simple to manufacture. However, they lack the ability to control moisture absorption from the wound, often leaving it too dry for timely healing. In the case of excessive wound drainage, traditional dressings can become adhesive to the wound, leading to complex and painful dressing removal. Wet-to-dry bandages can also cause vasoconstriction, hypoxia, and further tissue damage during removal. Many traditional dressings are also susceptible to bacterial contamination, which can hinder the healing process. The limitations and advantages of traditional dressing are shown in Figure 2.

### 3.2. Topical Formulations

Topical formulations are another category of traditional systems, such as gels, ointments, and creams. Many commercial formulations are available, such as chlorhexidine, povidone iodine, hydrogen peroxide, and cadexomer iodine [44,50]. Chlorhexidine is used to wash wounds due to its antibacterial and antiseptic properties as one of the main components in wound healing, especially in burn units of hospitals. However, some complications, such as its effects on healing, re-epithelialization, and cytotoxicity, are still contradictory [51,52]. Povidone iodine, available in various forms (such as solution, cream, etc.), is considered suitable for wound healing due to its extensive antimicrobial spectrum and efficacy on inflammation regarding lack of antibacterial resistance but the main drawback of povidone iodine is cloth staining [53]. Hydrogen peroxide is used as a disinfectant to clean the wound. Studies indicated positive effects of hydrogen peroxide, such as stimulation of induction of fibroblast proliferation and collagen, as well as negative attributes such as cytotoxicity, induction of apoptosis, and scarring on the wound location [54]. Cadexomer iodine exhibits dual functionality, encompassing wound cleansing and bactericidal action, while simultaneously facilitating fluid absorption and debridement. Notably, it lacks the cytotoxic effects attributed to another iodine-based preparation [55]. In contrast, low-viscosity formulations such as solutions exhibit limited efficacy in promoting wound healing, mainly due to their brief duration of stay at the wound site [44]. Semi-solid formulations like silver sulphadiazine cream and silver nitrate ointment, employed for the purpose of combating bacterial infections, exhibit an enhanced residence time on the wound surface in contrast to solution-based counterparts. However, in cases involving exudative wounds of pronounced magnitude, the efficacy of semi-solid preparations declines significantly, attributable to their swift assimilation of bodily fluids, subsequent loss of rheological attributes, and consequent acquisition of mobility [44]. Because of the limitations and shortcomings of the old wound healing systems, a notice of the need for novel wound dressing and healing systems has emerged.

## 4. Novel Wound Dressing Technology and Different Platforms

### 4.1. Novel Wound Care Management

Due to the aforementioned particular properties of the ideal wound dressing, despite millennia of wound healing process description and seeking dressing materials and devices, most available and traditional dressings cannot conveniently meet all of the requirements. Modern wound dressings are designed not only to cover wounds but also to promote the wound-healing process. Since the 1960s, many advanced dressings have been created and showcased for their superior properties. Major categories of the aforementioned modern dressings include films, foams, hydrogels, natural polymeric fibers, tissue-engineering technology-prepared dressings, and electrospun fiber dressings. These dressings are designed to prevent wounds from losing moisture and boost the healing process [56,57,58,59].

#### 4.1.1. Films

Films, also called semipermeable films, were one of the first advances in wound care [47]. Films are polyurethane or copolyester sheets on an adhesive layer [60]. The physical characteristics of these films include a porous and thin structure, which supplies proper gas permeability but exhibits poor water permeability and impermeability to bacteria; translucency; self-adhesion; and flexibility, which makes them a good choice for flexor and extensor anatomical areas. Semipermeable films can be used as secondary dressings and primary dressings (e.g., foams) to make them waterproof and to deal with the films’ shortcomings in exudate absorption [47,61]. Another problem with using film dressing, besides poor exudate absorption, is the possibility of epidermal damage and unwanted trauma because of their adhesiveness when removed from the area [62]. Layer-by-layer self-assembly techniques generally prepare films. In these approaches, the spontaneous deposition of oppositely charged ions on solid texture by alternating exposure to the solutions of positively and negatively charged polymers produces thin-layer films. Two or more layers based on the required properties and characteristics can be achieved with this method [61].

#### 4.1.2. Foams

The foam dressings are manufactured using porous polyurethane or a semi-occlusive silicone center on a sheet or semi-solid structure with or without an adhesive layer and exhibit high absorption capability, debridement promotion, and gas and water vapor permeability [57,63,64]. Foam dressings are commonly used in wounds with moderate-to-heavy exudation to provide a moist environment to promote healing. The drawbacks of foam dressings are that they need to be changed frequently and are unsuitable for low-exuding or dry wounds because recovery depends on exudate when treated with foam [58]. Various approaches are used to prepare desirable foams. They can be obtained by the simple interaction of ingredients such as polyphenols with diisocyanate or with lyophilization and other simple methods, which could be mentioned as another advantage for this category of novel dressings [65,66].

#### 4.1.3. Hydrogels

Maintaining moisture in the wound site is one of the well-established requirements for achieving boosted wound healing. Hydrogel-based dressings, as an example of the novel-developed dressings, with high water content in their structure, were developed to meet this requirement [58]. Synthetic and biopolymers are crosslinked to prepare insoluble hydrophilic hydrogels with special attributes of physical structure, indicating good exudate absorption and moisture and gas transmission. However, as a shortcoming, the low physical strength of hydrogels impacts their application as a single material, and thus, they are usually hybridized with other platforms [67]. The methods used to obtain hydrogels generally include using chemical crosslinkers to connect the polymers used and solvent casting at room temperature, in an incubator, or through lyophilization [68,69,70].

### 4.2. Biopolymers in Wound Care

The application of several biopolymers in developing novel wound dressings has been examined for years. Collagen, hyaluronic acid, chitosan (CS), and alginate were the best candidates in this process. Alginate-based dressings appear to outperform the competition and are already being marketed as wound-care materials [71]. Alginate is a natural linear anionic polymer that is obtained from brown seaweed, incorporating D-mannuronic acid and L-guluronic acid subunits. Gel formation by ion exchange between calcium in alginate and sodium of exudate made this dressing a proper agent for medium to highly exuding wounds. But in little or no exudate, this gel formation with healthy tissue damages the skin [59,71]. In summary, good fluid absorption capacity and biocompatibility made alginate dressings and all-natural polymeric fibers adequate wound dressings.

Over the past few decades, tissue-engineered dressings have been used to imitate the natural scaffold and matrix of wound-healing areas. However, conventional bandages cannot replace lost tissue in serious injuries. To fill this gap, human skin equivalents (HSEs) have been developed to accelerate wound healing and replace lost tissue [57]. HSE is commonly made up of keratinocytes and fibroblasts within extracellular matrix ingredients (regularly collagen), human placenta, or acellular matrix. Some FDA-approved products of tissue-engineered dressings are available and can be acquired in medical and pharmaceutical markets [72]. For example, Apligraf represents an intricate composition comprising a bovine collagen matrix intermingled with a stratified human epithelial layer, wherein reside metabolically active fibroblasts within the dermal component and proliferating keratinocytes within the epidermal component [73].

### 4.3. Bioprinting Techniques

Bioprinting techniques have emerged as a transformative approach in the development of multilayered wound dressings, enhancing healing processes through biomimetic structures. Bioprinting techniques for multilayered wound dressings involve various strategies, like coaxial bioprinting and multi-material bioprinting, to create complex structures. These techniques allow for the precise fabrication of skin substitutes that replicate the natural architecture of the skin, facilitating improved integration and functionality [74,75]. Bioprinting employs a diverse array of materials, including live cells, hydrogels, and other biomaterials, to fabricate multilayered constructs [76,77]. These constructs, such as microfragmented adipose extracellular matrix (mFAECM), enhance cellular adhesion and expedite the wound healing process. Advanced 3D-bioprinted biomimetic scaffolds, incorporating mFAECM and cellular components, accelerate tissue repair by promoting tissue contraction, collagen deposition, and neovascularization in full-thickness dermal defects [78]. Hydrogel-based wound dressings, designed in multilayered configurations to replicate the native cellular microenvironment, significantly improve healing outcomes. 3D-printed biocomposite hydrogels can be functionalized with therapeutic agents (e.g., silver nanoparticles, growth factors) to enable controlled release, enhancing granulation tissue formation and increasing vascular density [76]. Recent studies have demonstrated that bioprinted skin models, constructed using multilayered hydrogel scaffolds, successfully induce capillary network formation. This vascularization is essential for nutrient diffusion and waste clearance, critical for the survival and function of skin cells [79]. Bioprinted constructs can mimic the complex architecture of the epidermis, dermis, and hypodermis, promoting rapid vascularization and normal extracellular matrix synthesis in vivo. Research indicates that bioprinted skin grafts significantly enhance epithelialization and mitigate fibrosis, representing a substantial advancement in wound repair methodologies [77,79]. Despite these promising developments, challenges such as biomaterial sourcing and the time required for fabrication remain significant barriers to the broader clinical implementation of bioprinting technologies.

### 4.4. Electrospinning Technology

Wound dressings manufactured using electrospinning technology deserve special mention due to their high surface area-to-volume ratio, porosity, and capability of loading drugs and bioactive agents. This technique allows for the creation of nanofibrous membranes that can be tailored to meet specific therapeutic needs [80]. In this technique, a high electrical potential is generated between two electrodes of opposite polarity to overcome the surface tension of the polymer solution and evaporate the solvent, producing fibers [81]. They can be used singularly as a wound dressing in combination with drugs or in tissue engineering.

Electrospinning facilitates the incorporation of diverse biomaterials, including polycaprolactone and collagen, to fabricate advanced wound dressings with enhanced moisture retention and antimicrobial efficacy. The integration of electrospun nanofibrous matrices with additional biocompatible materials, such as gelatin and chitosan, generates composite dressings that not only support cellular adhesion and proliferation but also establish an effective barrier against microbial infiltration [82,83,84]. The highly porous architecture of electrospun nanofibers closely emulates the structure of the extracellular matrix, promoting superior integration with the wound environment and improving biocompatibility. Multilayered dressings can be engineered to incorporate antimicrobial agents, such as silver nanoparticles, providing infection control while concurrently supporting tissue regeneration [85,86]. This method was developed to overcome the waste of cost, time, and material in other methods [87].

The upcoming discussion focused on multilayered or composite dressings produced by novel technologies.

## 5. Different Processing Methods

### 5.1. The Electrospinning Processes

With a closer look at major processing methods of novel wound dressing platforms, they could be classified into four main groups including electrospinning, layer-by-layer casting, freeze-drying, and layer-by-layer self-assembly.

**Figure 3:**
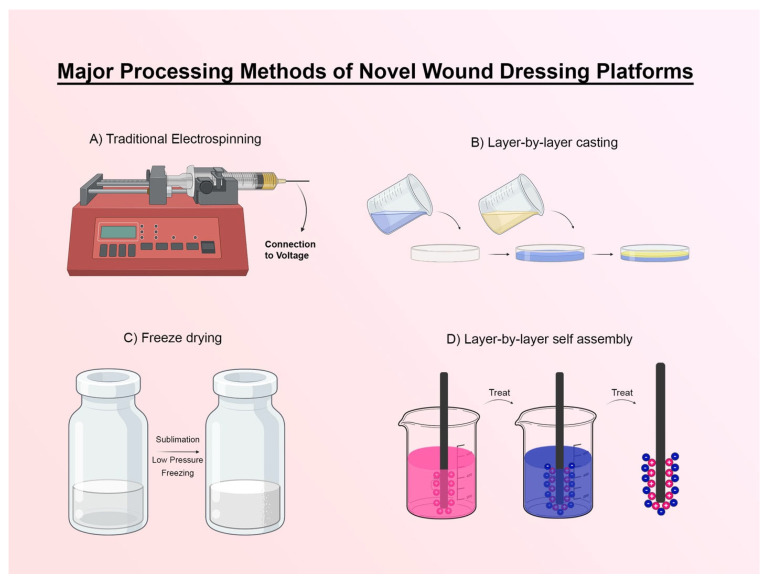
Major processing methods of novel wound dressing platforms: (**A**) Traditional electrospinning device, (**B**) schematic of layer-by-layer casting (solvent casting) method, (**C**) schematic of freeze-drying, and (**D**) schematic of layer-by-layer self-assembly method.

In the electrospinning process (Figure 3A), there were two main approaches mostly used in recent articles of multilayered wound dressings, including common or single nozzle, and sequential which includes the two-nozzle approach. Both approaches are divided into subtypes based on the shapes and angles of needles in contrast to each other. In a recent study, a single approach with syringes with a blunt-end metal capillary nozzle and a receiving medium of a simple foil layer was adopted to achieve two layers: one antibacterial layer (ABL) from the treated solution of zein, Ethylcellulose, and Vaccarin (an herbal extract that hastens wound healing) as the top layer and one healing promotion layer (HPL) from the treated solution of zein, Ethylcellulose, and Protoporphyrin IX (an analog of Porphyrins that acts as a mediator in the wound healing process) as the bottom layer [23,88,89]. A membrane as a reinforcement layer (RFL) is used as the middle layer to mainly stabilize these layers. Engaging in a dynamic agitation process, wherein reactants are meticulously combined with a precisely calibrated concentration of ingredients, gives rise to the creation of a homogeneous solution, thereby constituting the step of solution treatment, exemplifying the art of optimization [23]. In another study, the two-nozzle electrospinning method with a drum collector was used in a further described manner. Polycaprolactone (PCL) loaded with curcumin solution from the first nozzle was electrospun for a while to prepare the first layer, then concurrently, the second nozzle started the process of injection of polyvinylalcohol (PVA) solution. Finally, the second nozzle stopped and the first nozzle kept working PCA for a while of time. The manipulation of base solutions entails the application of heating, the induction of mechanical agitation, and the dissolution of solutes within a compatible solvent matrix [90]. Conventional electrospinning is an effective and versatile method employed for fabricating nanofibers. However, the relatively low production rate is the major challenge of electrospinning as an economic and scalable method. Recently, several approaches have been developed to ensure the high production rate of nanofibers, such as multi-jet, needleless, bubble, centrifuge, and air-sealed centrifugal electrospinning [91]. All the mentioned techniques have been developed in order to obtain nanofibers with better quality and a high production rate. Due to the focus of the present article on the techniques used in the articles of the last 5 years, only a brief mention of more advanced techniques is sufficient.

### 5.2. Solvent Casting

Layer-by-layer casting (solvent casting) is one of the simplest approaches to attain layered platforms (Figure 3B). A paper on layered dressing with a releasing profile prepared a four-layered structure one by one, casting each layer on the previous one in a Teflon petri dish [92]. It is worth mentioning that, as a branch of the basic technique of layer-by-layer casting, the spin-coating technique is also introduced. Spin coating is a widely utilized method in material science for fabricating thin films on substrates through the deposition of liquid material, typically a polymer solution, onto a rotating substrate [93,94]. The centrifugal force spreads the liquid uniformly across the surface, creating a thin film whose thickness and uniformity are controlled by the spinning speed and duration [95]. In the context of bilayered membrane fabrication, materials like gelatin and polycaprolactone (PCL) were used, with PCL forming the base layer of a wound dressing. The process involves preparing the substrate, depositing the solution, spinning it under atmospheric conditions, and drying or curing the film, often using crosslinking polymerization to enhance membrane properties. Spin coating offers advantages such as uniform film formation, scalability for industrial applications, and versatility in material selection, making it ideal for biomedical uses like antibacterial and cytocompatible wound dressings [96].

### 5.3. Lyophilization

Vakilian S. et al. used lyophilization to prepare a loading layer for herbal extract. Lyophilization involves the removal of water from a material through sublimation, which occurs when ice in its solid form is transformed directly into vapor under reduced atmospheric pressure (Figure 3C). This method can result in a highly porous structure and make it suitable for loading drugs or reaching high permeability [97,98].

### 5.4. Layer-by-Layer Self-Assembly Method

Layer-by-layer self-assembly (electrostatic or pH-amplified self-assembly), or in some cases known as dip coating, consists of immersing a base in solutions of ingredients with opposite electric charges with a period of heating or a while of time to dry [17]. In this method, it is possible to reach a high number of layers, including 8, 15, and even hundreds of layers, which can have different uses depending on the intended function (Figure 3D).

## 6. MultiLayered Dressings (Composite Dressings)

**Figure 4:**
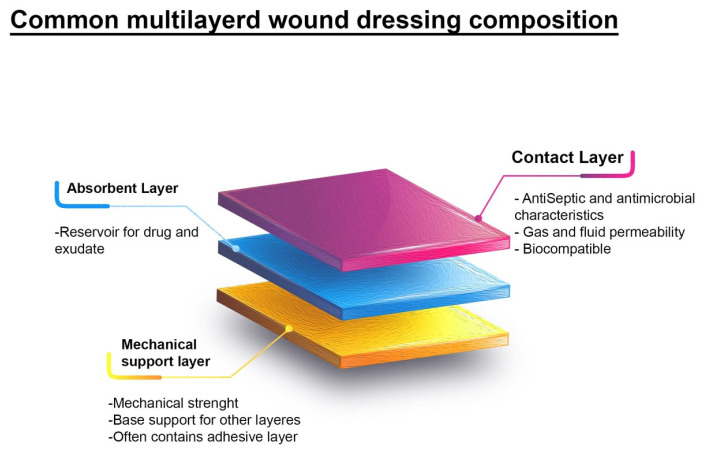
Common multilayered wound dressing composition. This scheme shows a common tertiary layered dressing, which includes contact layer, absorbent layer, and mechanical support layer, respectively.

Recently, the novel single-layer dressings have been abandoned, and multilayered dressings are put at the center of attention so that all or most of the required and desirable features mentioned in the previous sections can be achieved with only one type of dressing. In instances of most monolayer platforms, the exhibition of drug release profiles manifests as rapid and intense bursts. This particular drug release profile imparts constraints upon wound healing procedures, particularly when confronting wounds characterized by heightened infection rates, thereby necessitating a greater assurance of dressing reliability and an extended duration of stay within the wound site. Typically, a sustained and protracted release regimen over an extended timeframe is contemplated as a more desirable strategy, given its propensity to engender superior therapeutic outcomes across a vast array of bioactive agents. This issue directs research to come up with structures comprised of more layers with controlled release properties, and further, such as providing antibacterial properties, improving healing, and special mechanical properties (to be placed in the outermost position from the wound) in different layers’ composition properties (Figure 4).

All the research of the last 5 years, according to the proposed method in the field of multilayer dressings, has been fully analyzed in Table 1, and the special features of each case have been presented. The literature surveyed reveals the prevalent existence of assorted and arbitrarily named wound dressing platforms. The categorization of these compounds lacks specific guiding principles, as the sources do not provide explicit criteria for classification. Instead, previous studies, synthesis methods, and preparation techniques serve as the basis for classification. Multilayer platforms, proposed to offer numerous benefits, encompass features such as notable porosity, favorable water vapor permeability, robust mechanical strength, cost-effectiveness, potent antimicrobial and anti-inflammatory properties, the absence of toxic chemical initiators and crosslinkers (e.g., gamma rays as an alternative to chemical crosslinkers), and acting as carriers for the controlled and consistent release of plant extracts, drugs, or bioactive factors. These advantageous characteristics resemble those found in commonly used traditional wound dressings. However, it remains challenging to incorporate multiple features simultaneously into a single-layer wound dressing. A cursory examination of the materials utilized in the fabrication of these wound dressings reveals a prominent usage of natural polymers and polysaccharides. Examples include silk cocoons of B. mori, gelatin, sodium alginate, konjac glucomannan, and chitosan. Incorporating natural materials not only facilitates accessibility and reduces costs but also imparts biocompatibility to these platforms, greatly assisting in their design. As for bioactive or pharmaceutical components, antibiotics such as gentamicin, ampicillin, and moxifloxacin have been employed. Nonsteroidal anti-inflammatory drugs (NSAIDs) like diclofenac, possessing anti-inflammatory properties, have also been utilization. Silver-based compounds have been chosen as additional active pharmaceutical ingredients (APIs). Additionally, natural substances, including ethanol extracts of propolis (EEP) and probiotics like Lactobacillus brevis, have been incorporated due to their diverse range of activities, including anti-inflammatory and antimicrobial effects. Toxicity evaluations of these platforms have been conducted through in vivo and in vitro tests. Mouse fibroblast cells (L929), normal human dermal fibroblasts, and, in some instances, human keratinocytes (HaCaT) have been employed to assess cytotoxicity. Animal tests have utilized full-thickness skin defect wound models, created either surgically or through burning, occasionally inducing infection.

## 7. Hemostatic Agents

The concept of homeostasis pertains to the body’s remarkable ability to maintain a stable internal environment notwithstanding external fluctuations [123]. It plays a pivotal role in ensuring the proper functioning of cells and tissues. In the context of wound healing, homeostasis assumes immense significance as it governs the regulation of inflammation and guards against bacterial colonization, thereby fostering optimal healing [124]. To this end, the integration of hemostatic agents within wound dressings proves instrumental in establishing a conducive milieu for wound healing. By ensuring a stable environment within the wound bed, these agents stimulate healing processes while warding off infection. In exigent circumstances like war zones and emergencies, hemostatic agents become indispensable for saving lives when the body’s inherent clotting mechanisms prove inadequate. Notably, the incorporation of hemostatic agents into multilayered wound dressings has exhibited promising outcomes in enhancing wound healing outcomes. The diverse array of hemostatic agents and materials encompasses both biologically derived and synthetic polymers, as well as minerals such as chitosan, collagen/gelatin, alginate, oxidized cellulose, dextran, hyaluronic acid, starch, aliphatic polyesters like polycaprolactone (PCL) and polylactic-co-glycolic acid (PLGA), polyethylene glycol (PEG), polycyanoacrylate, polyurethane (PU), kaolin, silver, copper, zinc, and zeolite. These aforementioned materials have been integrated into or developed as multilayered wound dressings with the aim of promoting hemostasis and curtailing further blood loss [125].

An ideal hemostatic material necessitates three crucial attributes: prompt and efficient cessation of bleeding, biodegradability, and compatibility with human tissue. Furthermore, it should boast commendable wound dressing properties, including blood absorption capacity and infection prevention [126]. Some substances derived from biological sources have been associated with reports of toxicity and disease transmission [127]. Yet, considering the numerous benefits elucidated in preceding sections, the FDA recommends the adoption of precautionary measures to avert any potential harm or illness associated with these substances. Such measures encompass a comprehensive characterization of the substance and assessment of its compatibility with living tissue. Rigorous in vitro testing and subsequent in vivo evaluations are indispensable for appraising its performance [128]. We propose that employing these compounds within multilayered structures, in accordance with synthetic methodologies, has the potential to mitigate the aforementioned issues, particularly toxicity concerns even at similar concentrations, while simultaneously enhancing physical properties.

## 8. Toxicological Issues Associated with MultiLayered Dressings

In addition to the other characteristics, a desirable wound dressing should be comprised of non-toxic, non-antigenic, biocompatible, and biodegradable materials [125,129,130,131], so that it does not release toxic products or cause adverse reactions. Thus, in vitro cytotoxic assays are utilized [132]. Also, acceptable scaffold biocompatibility is mandatory to achieve proper in vivo performance [133].

Studies regarding the in vitro safety of multilayer wound dressings demonstrate that although the addition of pharmaceutical ingredients may provide the desired antimicrobial properties, the toxicity of this proportion may also cause harm to the wound tissue. For example, curcumin-loaded PCL/PVA multilayer nanofibrous dressings show antibacterial effects, but exceeding 16% of curcumin exhibits approximately 60% of cell survival after 48 h in the L929 cells [90]. In another work, it has been shown that chitosan-chelated nano-level silver ions increase the antibacterial potential of the dressings. However, in higher concentrations, cytotoxicity is observed. In order to avoid toxicity while ensuring the proper delivery of a therapeutic dose to the wound, some structural improvements, like the acetal reaction in alkaline solution to produce silver chitosan/polyvinyl alcohol sponge [134,135], or dressings providing controlled/sustained release of the active ingredient have been investigated [136]. It is also addressed that in order to achieve selectivity for binding specific biomolecules while loading proteins/enzymes on LbL films, techniques like the application of embedded complexes of Ni2+ to adsorb histidine6-tagged proteins have been investigated [137,138], and from the toxicological perspective, the possibility of Ni2+ leakage to the outside of the multilayer matrix and potentially harming the living organisms around proves challenging [139].

The polymers used in wound dressings are either synthetic polymers or natural polymers. Synthetic polymers, e.g., poly (lactic-co-glycolic acid), PCL, PVA, PEG, PLA, and PLLA, are shown to be non-toxic in biology. However, they lack cell-binding sites. On the other hand, natural materials such as gelatin, collagen, cellulose, chitosan, and SF, conserving the advantages of synthetic polymers regarding biocompatibility, contain biological sites on their surface which are recognized explicitly by cell integrins and can result in elevations in cell adhesion, migration, and proliferation, and eventually enhanced tissue reconstruction [140,141].

In layered systems/dressings, in order to obtain nanofibers, some harmful and toxic organic solvents, such as (usually 2,2,2-trifluoroethanol (TFE), trifluoroacetic acid (TFA), and 1,1,1,3,3,3-hexafluoro-2-propanol (HFIP)), are utilized, and solvents remaining in the fiber may affect the subsequent use due to their toxic or corrosive nature. However, techniques like using an acidic solvent, e.g., acetic acid and aqueous formic acid, in the electrospinning solution are currently applied to avoid toxic solvents [140,142]. Furthermore, the electrospinning of environmentally friendly and non-toxic polymers and solvents, known as green electrospinning, is an important idea in pharmaceutical products [143,144]. Therefore, this approach has been of great interest [145,146,147]; hence electrospinning technology allows us to fabricate ultrafine fibers utilizing different setups. In this regard, either solution (e.g., blend, coaxial, and emulsion) electrospinning or green solvent-free electrospinning procedures can be used in producing biocompatible fibers, especially for wound care [148].

In a recent animal study of Oligomer Chitosan/Polyvinylpyrrolidone Coated dressings, it has been demonstrated that the release of a high amount of Oligomer Chitosan (COS) from COS6, i.e., the spraying solution of 3% COS on the PCL base, dressings can generate unfavorable effects on skin regeneration in mice [149], while PVP6–6, i.e., 6% COS and 6% PVP, as well as COS3 and PVP6–3, facilitate the process, which can be attributed to the C=O groups of PVP acting as proton-acceptors that interact with amine groups of COS and lower the impact of COS on the tissue [144,150]. In another study, PCL/COS16 displayed excellent antibacterial performance, but in contrast to the low COS dose samples, negative results in the case of biocompatibility and in vivo wound healing were observed in mice. This work reported the dressing with PCL/COS8 as the optimal sample [149]. Moreover, it has been mentioned that low-chitin and high-chitin materials at high concentrations are toxic to fibroblasts [151].

Due to their direct attachment to the wound bed, it is also a primary prerequisite for hydrogel-based wound dressings not to be toxic and immunogenic [152,153,154,155,156]. Moreover, in biodegradable hydrogels, the fragments produced after degradation of the material should be safe. Using high-purity polymer solutions along with biocompatible procedures, for instance, harmless UV conditions, are also some fundamental matters to avoid toxicity [157,158].

Crosslinkers are other components of electrospun dressings whose biocompatibility is of great importance. As an example, while using glutaraldehyde as the crosslinker, cell viability and cell proliferation are negatively affected [31,159,160,161]. Furthermore, in the study of a bilayer scaffold composed of electrospun polycaprolactone and poly (lacto-co-glycolic acid) (PCL/PLGA) membrane and glutaraldehyde (3.5% *v*/*v*) crosslinked chitosan/gelatin hydrogel, the addition of a hydrophobic membrane layer along with the effect of glutaraldehyde crosslinking has been concluded to cause a decline in cell viability, which could be prevented by washing with glycine to neutralize the crosslinker [159]. genipin, being less toxic than glutaraldehyde, is a natural compound with two functional groups reacting with amine groups, making this compound likely to be used as a natural crosslinker for proteins, collagen, gelatin, and chitosan. It has been exposed that genipin crosslinked CHI/HA and CHI/ALG LbL films significantly affect cell adhesion behavior [139,162].

Cytotoxicity and cell attachment assays have demonstrated that free-standing CHI-ALG films represent a poorly adhesive substratum for human dermal fibroblasts. On the other hand, no signs of cytotoxicity were detected in a trial of 7 days while plastic-adherent fibroblasts were incubated beneath floating films [163]. Also, because of the non-significant decline in FI and the closeness of the values of genipin, native, and CaCl_2_ crosslinked films, cytotoxic effects are negligible [163].

Sodium alginate (SA) is an anionic polysaccharide that can be crosslinked via bivalent ions like Ca^2+^ and Zn^2+^ so that the high toxicity of chemical crosslinkers like glutaraldehyde and epichlorohydrin can be avoided [164,165]. Also, this reaction can occur under very mild conditions at room temperature and without toxic solvents [166]. Besides other advantages, the non-toxicity, biocompatibility, and hemostatic properties make alginate gels a prevalently used material in fabricating wound dressings [167,168,169].

## 9. Conclusions

In conclusion, the domain of wound care has experienced substantial advancements, particularly through the development of novel multilayered wound dressing technologies that hold promise for enhancing healing outcomes in both acute and chronic wounds. The development of advanced wound dressing platforms has emerged as a promising strategy to address the limitations of traditional wound management approaches. Multilayered dressings, in particular, offer a versatile solution by integrating diverse functionalities within a single system. These composite structures leverage the unique properties of various materials to create an optimal microenvironment for accelerated wound healing. Nonetheless, several challenges still persist that demand further studies. Future research should focus on the development of novel biocompatible and biodegradable materials, as certain synthetic polymers can elicit cytotoxic responses; therefore, it is imperative to explore the utilization of green electrospinning techniques, natural polymers, and less toxic crosslinking agents. The challenge of achieving controlled drug release is also notable, with many dressings exhibiting rapid burst release profiles; developing sophisticated release mechanisms, including electrospun nanofibers or microencapsulation strategies, may facilitate more sustained therapeutic delivery. Furthermore, the scalability and cost-effectiveness of the production processes for these advanced wound dressings remain a concern, hindering their widespread adoption in clinical settings. Addressing these challenges will necessitate interdisciplinary collaboration among research, clinic, and industry. Ongoing advancements in material science, biotechnology, and engineering will be crucial for the development of next-generation wound dressings. Furthermore, the integration of smart technologies, such as biosensors for real-time monitoring of wound conditions, could significantly enhance treatment strategies. Another area of focus should be the incorporation of smart, responsive features into multilayer wound dressings, enabling them to dynamically adapt to the changing wound microenvironment. This could involve the development of dressings with the ability to sense and respond to specific biochemical cues, triggering the controlled release of therapeutic agents or the modulation of physical properties to better support the healing process. Additionally, the personalization of treatment approaches is often inadequate, as many dressings adopt a one-size-fits-all model; customizing wound dressings based on individual patient characteristics could optimize healing outcomes, potentially employing technologies such as 3D printing for tailored solutions. Furthermore, the integration of advanced bioprinting and tissue-engineering strategies into the design of multilayer wound dressings holds immense promise. By precisely replicating the complex skin architecture and cellular microenvironment, these biofabricated constructs could significantly enhance wound healing outcomes, particularly in the context of severe, full-thickness injuries. By concentrating efforts on these areas, the field of wound care can markedly improve patient outcomes and mitigate the burden associated with chronic wounds on healthcare systems globally.

## Figures and Tables

**Table 1:** Platforms and processing methods in wound dressing agents.

Platform	Claimed Advantages	Material	API/Uniqueness	Toxicity Tests	In Vivo/ In Vitro	Type of Injury	Brief Explanation	Ref.
Bilayer hydrogel	Without using toxic chemical initiators and crosslinkers, freely variable thickness of layers due to high penetration of γ-rays, both wound dressing and anti-adhesion capabilities	Gelatin Polyvinyl alcohol (PVA)	Peritoneal adhesion reduction	Cell culture of 3T3-Swiss fibroblasts	Cell line	-	This bilayer hydrogel of Gelatin and PVA formed by radiation-induced crosslinking with γ-rays	[99]
Bilayer sponges	Water vapor permeability, antimicrobial, and anti-inflammatory effect	Polyhedral Oligomeric Silsesquioxane (POSS) Chitosan	Cissus quadrangularis (CQ) extract	Cell culture of 3T3-Swiss fibroblasts	Cell line	-	upper layer was prepared using CQ extract-loaded chitosan nanospheres and nanofibers prepared by electro-spraying and electrospinning techniques, respectively. Subsequently, chitosan/POSS mixture lyophilized to shape sponges, which stand as the bottom layer. Finally, a last electro-spraying stage performed to coat upper layer on bottom layer.	[100]
Composite bilayer porous film	Good water vapor permeability, very long-lasting, broad-spectrum antibacterial properties	Graphene and Graphene Oxide (GO) thermoplastic polyurethane (TPU)	Polyhexamethylene guanidine hydrochloride (PHMG)	human epidermal keratinocyte line (HaCaT) Full-thickness skin defect infected wound model on BALB/c mice	Cell line and animal study	Full-thickness skin defect infected wound model	PHMG is covalently grafted to the GO surface by nonsolvent phase separation and particle filtration method, and MGO prepared a porous membrane with a skin-like thermoplastic polyurethane (TPU).	[101]
bilayer hybrid bio-scaffold	Low densities, high porosities, strong water absorption, and good mechanical strength, low cost	Ovine tendon collagen type I Palm tree-based nanocellulose	Biodegradability	-	-	-	Scaffolds fabricated from two different distinct layers of collagen and OPEFB nanocellulose powder were pipetted into a mold followed by the freeze-drying process.	[102]
Bilayer membrane	Improved bioavailability, maintenance of therapeutic concentration of drug, and avoidance of hepatic first-pass metabolism	Zein	gentamicin sulfate (GS)	NIH/3T3 fibroblast HS2 human dermal keratinocyte	Cell line	-	Zein as a film layer was fabricated by the solvent casting method to produce upper layer. The second zein layer loaded with gentamicin sulfate (GS) was used as a contact layer, prepared by sequential electrospinning method.	[103]
Bilayer scaffold	Good mechanical properties, remarkable biocompatibility, suitable water vapor transmission rate, appropriate anti-infective behavior, cell adhesion, and proliferation	The swine’s small intestine submucosa (SIS)	Great amounts of collagens and wide variety of cytokines with low immunogenicity	Full-thickness skin defect wound model on C57/BL mice	Animal study	Full-thickness skin defect wound model	SIS powder dispersed solution pipetted into a mold with an SIS membrane on bottom and was frozen to prepare a bilayer of cryogel/membrane dressing.	[104]
Electrospun nanofibrous patches	carrier for plant extract delivery with controlled and sustained release characteristics, improves physical properties of the wound dressing, and time-programmed multi-agent release	Poly (ε-caprolactone) (PCL) Carboxyethyl chitosan (CECS) Polyvinyl alcohol (PVA)	Chamomile extract as an antioxidant/antibacterial agent	Adipose-derived mesenchymal stem cells (AdMSCs) (MTT assay test)	Cell line	-	Multilayer patches with a contact layer composed of chamomile loaded CECS/PVA nanofibrous and a PCL nanofibrous layer to provide mechanical strength. An intermediate layer made of PCL and chamomile/CECS/PVA prepared as cohesion promoter with two-nozzle method between the two previous layers with two-nuzzle electrospinning method.	[105]
Bilayer nanocapsule Dressing	Good biocompatibility, satisfactory cellular uptake, capacity, accelerated the epithelialization of the wound, reduced the levels of ROS and TGF-β, satisfactory wound-repairing and anti-scarring effects	cerium oxide (CeO_2_) pirfenidone (PFD) polylactic acid (PLA)	cerium oxide (CeO_2_) nanoparticles as ROS scavenger and pirfenidone (PFD) as anti-fibrotic	Normal human fibroblasts (NHF) Full-thickness skin defect wound model on Sprague–Dawley (SD) mice	Animal study	Full-thickness skin defect wound model	Nano-capsules (NCs) prepared with techniques of Layer-by-layer self-assembly method resulting in a core/shell (featuring PFD at their core and CeO_2_ in their shell). To achieve a dressing form, PFD/CeO_2_ NCs were fixed on plasma-etched polylactic acid (PLA) fiber membranes fabricated separately by electrospinning.	[106]
Multilayer coating covered textile	controlled release properties	non-woven polyethylene terephthalate (PET) Chitosan cyclodextrin	protective coating that limits the diffusion of silver and its side effects in wound spot without losing its biocide properties	-	-	-	PET textiles were pre-treated by chitosan and cyclodextrin with a pad/dry/cure method and thereafter were dipped in silver solution and in final stage were dipped in poly-cyclodextrin and chitosan to achieve a multilayer coat assembled in an electrostatic self-assembly manner. *	[107]
Free-standing multilayer films (FSF)	Reservoir for the sustained release	Chitosan Alginate	Fibroblast growth factor 2 (FGF2)	Normal human dermal fibroblasts Full-thickness skin defect wound model on mice	Cell line and animal study	Full-thickness skin defect wound model	An automated dip coater used to electrostatically assemble chitosan and alginate on a glass slide repetitively to achieve a 100-bilayer film. Finally, film detached and immersed in ginpin as a crosslinking agent. FGF2 loaded by incubating films in its solution overnight	[108]
Bilayer matrix	good absorption of wound exudates, cell adhesion, and cell proliferation	Collagen (fish of marine origin)	bioactive latex (L) from Calotropis procera	NIH 3T3 fibroblast Human keratinocyte (HaCaT)	Cell line	-	A spongy 3D matrix of collagen was fabricated with help of iterative freeze-drying. Then matrix placed on receiver part and cellulose acetate incorporated with latex or ciprofloxacin solution was electrospun over prepared sponge	[109]
Layer-by-layer coated textile	long-lasting antibacterial efficacy	polyethylene terephthalate (PET) Chitosan (CHT) Methyl-beta-cyclodextrin polymer (PCD)	chlorhexidine (CHX)	The human embryonic epithelial cell line (L132)	Cell line	-	Non-woven polyethylene terephthalate (PET) modified by using chitosan (CHT) and crosslinking process performed with genipin (Gpn). Methyl-beta-cyclodextrin polymer (PCD) and CHT deposited layer-by-layer on prepared textile with positive surface charge. Finally, CHX loaded in prepared structure.	[110]
BiLayer Scaffold	Proper chemical composition, thermal stability, wettability characteristics, and antibacterial activity as a drug delivery system, and also wound dressing system	Polyvinyl pyrrolidone (PVP) Gelatin (GEL) Cellulose acetate (CA)	Gentamicin	-	-	-	Sequential electrospinning method adopted to achieve a layer of cellulose acetate (CA) on a mixed layer of gelatin + polyvinylpyrrolidone. And finally, gentamicin loaded into the scaffold.	[111]
Layer-by-layer coated scaffold	Adjustable characteristics and drug carrier	Polycaprolcatone (PCL) Hyaluronic acid	Corneal wound dressing	-	-	-	Primary scaffold prepared by electrospinning PCL in different diameters. Then, surface modification by immersing fibers in chitosan preformed. PH-amplified coating performed once again by immersing fibers in an aqueous solution of hyaluronic acid or heparin.	[112]
Bilayer films	Better patient compliance with the drug delivery system, enhanced drug efficacy by sustained release behavior, and a decrement in adverse effects	Alginate Carboxymethyl cellulose (CMC)	Diclofenac	-	-	-	To prepare a bilayer film, a mixed 1:1 dispersion of alginate and CMC dried as simple casting in a petri dish in two steps. Diclofenac incorporation performed only on top layer.	[113]
Bilayer Wafer	drug delivery system, high fluid retention levels, bio-adhesive properties,	Gelatin Propylene glycol (PG) Methylcellulose (MC) Polyvinylpyrrolidone (PVP) Hydroxypropyl Methylcellulose (HPMC)	Moxifloxacin	Full-thickness skin defect wound model in BALB/c male mice	Animal study	Full-thickness skin defect wound model	The wafers were prepared by the lyophilization and casting method	[114]
Bilayer film	high biocompatibility, low cytotoxicity, and suitable mechanical and barrier properties	Chitosan Konjac glucomannan	Novel use of konjac glucomannan	-	-	-	Simply, subsequently two-step casting of chitosan and konjac glucomannan solution on a polystyrene plate prepared this film.	[115]
Bilayer hydrogel sponge/nanofiber	Bio_membrane mimetic structure, wound healing acceleration mediated with platelet-rich fibrin and L-arginine amino acids	Chitosan polyethylene glycol L-arginine	advanced platelet-rich fibrin (A-PRF)	Mouse fibroblast cells (L929) Full-thickness dorsal skin defect wound model on Wistar rats	Cell line and animal study	Full-thickness skin defect wound model	Chitosan/polyethylene glycol solution crosslinked and enriched with A-PRF and freeze-dried to make sponge as upper layer of dressing, and different percentages of L-arginine were mixed with Cs electrospun to prepare nanofibers as the bottom layer.	[116]
Hybrid bilayer hydrocolloid hydrogel	Thermally unstable probiotic delivery system, excellent mechanical properties	Sodium carboxymethylcellulose, polyisobutylene, styrene-isoprene-styrene, liquid paraffin, Polyvinyl alcohol, Chitosan, sodium alginate, hydroxypropyl cellulose	Lactobacillus brevis probiotic	Full-thickness Staphylococcus aureus-infected skin wound model on male Sprague–Dawley rats	Animal study	Full-thickness infected skin wound model	The hydrocolloid layer (bottom layer) was made from polyisobutylene (PIB) and styrene-isoprene-styrene block copolymer (SIS) using a hot-melt process. Also, different hydrogel layers (upper layer) were prepared by a freeze-thaw method using different combinations of PVA and various hydrophilic polymers to achieve desired characteristics on release profile.	[117]
Bilayer sponge scaffold	skin and soft tissue infections dressing, significant antibacterial effect against various strains, excellent delivery system	silk cocoons of B. mori, gelatin	CM11, a cationic antimicrobial peptide (AMP)	Human foreskin fibroblast cells (Hu02)	Cell line	-	Briefly fibroin/gelatin (SF/Gel) blend scaffolds loaded with various concentrations of a cationic antimicrobial peptide (CM11 peptide) and mixed and freeze-dried, following by a lyophilization stage to prepare bilayer sponge scaffolds.	[118]
Bilayer nanofiber	Good drug release kinetics and structure	Gelatin, sodium hyaluronate (HA), polycaprolactone (PCL), low molecular weight poly ethylene glycol	Ibuprofen	-	-	-	A hydrogel layer of gelatin (GE) and sodium hyaluronate (HA) film prepared as base and nanofibers of PCL/PEG fabricated by electrospinning and deposited on base layer via needleless technique. Also, Ibuprofen incorporated in PCL solution to achieve anti-inflammatory and pain-relieving properties.	[119]
Nanofibrous Scaffold	uniform morphology, bead-free structure of the PCL/Gel scaffold, antibacterial activity, high hydrophilicity, biodegradability, and biocompatibility	ethanolic extract of propolis (EEP), Polyurethane, Polycaprolactone (PCL), Gelatin	remarkable antibacterial activity against common wound infection bacteria due to presence of the top layer (PU/EEP)/the PU/EEP-PCL/Gel, significantly accelerated wound healing progression and shorter wound closure time	L929 mouse fibroblast cells Full-thickness dorsal skin defect wound model on female Wistar rats	Cell line and animal study	Full-thickness skin defect wound model	Polycaprolactone/gelatin (PCL/Gel) scaffold was electrospun on a dense membrane composed of polyurethane and ethanolic extract of propolis (PU/EEP).	[120]
Electrospun mat on foam	bead-free and uniform nanofibers with enhanced hydrophilicity, swelling ratio, degradation properties, and enhanced cell compatibility and healing properties	poly-ε-caprolactone (PCL), chitosan, ethanolic extract of propolis, polyurethane (PU)	Ethanolic extract of propolis (EEP)	L929 mouse fibroblast cells Full-thickness dorsal skin defect wound model on female Wistar rats	Cell line and animal study	Full-thickness skin defect wound model	The PCL/CS solution was electrospun on a PU foam coated with EEP to fabricate the PCL/CS-PU/EEP bilayer wound dressing.	[121]
Multilayer hydrogel	63% of the antibiotic was released after 7 days, antibacterial activity against oxacillin-sensitive S. aureus, no toxic effect on cultured fibroblasts,	carboxylated polyvinyl alcohol (PVA-C), gelatin (G), hyaluronic acid (HA) and gelatin	ampicillin (2 wt%) directly added to the HA solution	L929 mouse fibroblast cells	Cell line	-	ML hydrogels were prepared as four layers using carboxylated polyvinyl alcohol (PVA-C), gelatin (G), hyaluronic acid (HA) + ampicilin, and gelatin, respectively.	[92]
Bilayer hydrogel	complete re-epithelialization, fewer inflammatory cells, adherence to a large number of red blood cells and platelets, promoted blood coagulation and cell proliferation, antibacterial activity, excellent mechanical properties, good swelling, water retention, water vapor permeability, and biocompatibility	Polyvinyl alcohol (PVA), sodium alginate, chitosan, sodium tripolyphosphate, Pyridine-3, 5-dicarboxylic acid (H2PYDC), AgNO3	synergistic antibacterial action of the upper and lower layer, Ag@MOFs	mouse L929 fibroblastic cells	single horizontal diffusion cell/male BALB/c mice	full-thickness circular wounds	Ag-Metal−organic framework loaded chitosan nanoparticles (0.1%Ag@MOF/1.5%CSNPs) and polyvinyl alcohol/sodium alginate/chitosan (PACS) were used as the upper and lower layers	[122]

* The base method of preparing this platform is based on deep coating, but in the process, pad-dry-cure and electrostatic self-assembly methods can be observed.^.^

## Data Availability

No new data were created or analyzed in this study.
